# Broadly neutralizing antibodies against Omicron-included SARS-CoV-2 variants induced by vaccination

**DOI:** 10.1038/s41392-022-00987-z

**Published:** 2022-04-27

**Authors:** Xiangyang Chi, Yingying Guo, Guanying Zhang, Hancong Sun, Jun Zhang, Min Li, Zhengshan Chen, Jin Han, Yuanyuan Zhang, Xinghai Zhang, Pengfei Fan, Zhe Zhang, Busen Wang, Xiaodong Zai, Xuelian Han, Meng Hao, Ting Fang, Jinghan Xu, Shipo Wu, Yi Chen, Yingying Fang, Yunzhu Dong, Bingjie Sun, Jinlong Zhang, Jianmin Li, Guangyu Zhao, Changming Yu, Qiang Zhou, Wei Chen

**Affiliations:** 1 Institute of Biotechnology, Academy of Military Medical Sciences, Beijing, 100071 China; 2 Center for Infectious Disease Research, Westlake Laboratory of Life Sciences and Biomedicine, Key Laboratory of Structural Biology of Zhejiang Province, Institute of Biology, Westlake Institute for Advanced Study, School of Life Sciences, Westlake University, Hangzhou, 310024 Zhejiang Province China; 3 State Key Laboratory of Pathogen and Biosecurity, Institute of Microbiology and Epidemiology, Academy of Military Medical Sciences, Beijing, 100071 China; 4 Wuhan Institute of Virology, Center for Biosafety Mega-Science, Chinese Academy of Sciences, Wuhan, Hubei 430071 China

**Keywords:** Immunotherapy, Drug screening

## Abstract

The SARS-CoV-2 Omicron variant shows substantial resistance to neutralization by infection- and vaccination-induced antibodies, highlighting the demands for research on the continuing discovery of broadly neutralizing antibodies (bnAbs). Here, we developed a panel of bnAbs against Omicron and other variants of concern (VOCs) elicited by vaccination of adenovirus-vectored COVID-19 vaccine (Ad5-nCoV). We also investigated the human longitudinal antibody responses following vaccination and demonstrated how the bnAbs evolved over time. A monoclonal antibody (mAb), named ZWD12, exhibited potent and broad neutralization against SARS-CoV-2 variants Alpha, Beta, Gamma, Kappa, Delta, and Omicron by blocking the spike protein binding to the angiotensin-converting enzyme 2 (ACE2) and provided complete protection in the challenged prophylactic and therapeutic K18-hACE2 transgenic mouse model. We defined the ZWD12 epitope by determining its structure in complex with the spike (S) protein via cryo-electron microscopy. This study affords the potential to develop broadly therapeutic mAb drugs and suggests that the RBD epitope bound by ZWD12 is a rational target for the design of a broad spectrum of vaccines.

## Introduction

The pandemic of severe acute respiratory syndrome coronavirus 2 (SARS-CoV-2), the causal agent of coronavirus disease 2019 (COVID-19), has resulted in more than 280 million infections and more than 5.4 million deaths worldwide. During the pandemic, mutations in the SARS-CoV-2 genome have been accumulating continuously. As of December 2021, five variants of concern (VOCs) of SARS-CoV-2 have been announced by the World Health Organization (WHO), including B.1.1.7 (Alpha), B.1.351 (Beta), P.1 (Gamma), B.1.617.2 (Delta), and B.1.1.529 (Omicron).^1–6^ The Omicron variant contains an alarming number of mutations (almost 40) in its spike (S) protein (Supplementary Fig. 1) and has spread rapidly worldwide.^7^

The S protein on the Coronavirus surface recognizes the human membrane protein, facilitates the viral entry to the host cells, and thus constitutes the main target for neutralizing antibodies (nAbs). Three S1/S2 heterodimers are assembled to form a trimer S protein. S1 contains the N-terminal domain and the receptor-binding domain (RBD) that contacts with the host cell surface receptor protein, angiotensin-converting enzyme 2 (ACE2).^8,9^ The RBD adopts either “down” (also called “close”) or “up” (also called “open”) conformation, and the ACE2 can only bind to the RBD in the “up” conformation.^10^ NAbs play important roles in blocking viral infection and the clearance of viral particles. NAbs targeting RBD are characterized into six groups (group A to F) by Cao et al.^11^ Group A-D mAbs target the receptor-binding site (RBS) through different binding mode with various states of RBDs. Group E and F mAbs target more conserved epitopes outside the ACE2-binding site.

The ongoing evolution of SARS-CoV-2 variants raises concerns about the effectiveness of monoclonal antibody (mAb) therapies and potential evasion from vaccine-induced immunity.^2,4,5^ Recently, the reduced sensitivity of Omicron to several approved and clinical-stage mAbs and resistance to neutralization of plasma and sera elicited by vaccines were reported.^11–15^ The ongoing immune-escaping SARS-CoV-2 mutations highlight the urgent demands for broadly neutralizing antibodies (bnAbs).

In this work, we investigated longitudinal human plasma responses following the prime and the boost vaccination with the adenovirus-vectored COVID-19 vaccine (Ad5-nCoV, Convidecia), which has been approved for emergency use in over 10 countries,^16,17^ revealing the durability of nAb responses against SARS-CoV-2 VOCs, including Omicron. In addition, we developed a panel of bnAbs against Omicron and other SARS-CoV-2 VOCs and demonstrated their evolution over time. Cryo-electron microscopy (cryo-EM) structure determination revealed the structural basis of the nAbs with broad neutralization ability. This study reveals the potency of vaccine-induced bnAbs against current VOCs and affords the potential for broad therapeutic mAb drugs.

## Results

### Polyclonal antibody responses to vaccination

**Fig. 1:**
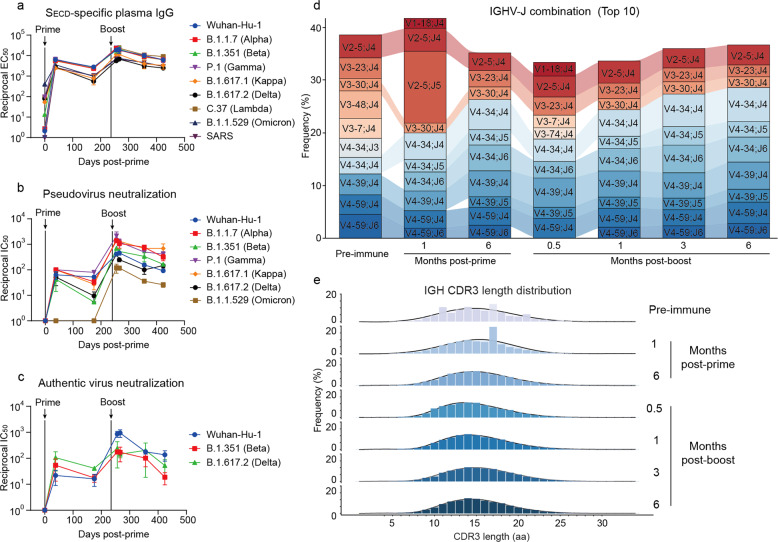
Polyclonal antibody responses to vaccination. **a** EC_50_ titers of binding IgG antibodies against S proteins of different SARS-CoV-2 variants over time in plasma samples. **b** Pseudovirus neutralization IC_50_ titers against SARS-CoV-2 variants in plasma samples. **c** Authentic neutralization IC_50_ titers of SARS-CoV-2 variants in plasma samples. The data are representative of one of at least two independent experiments and are presented as the mean ± SD. **d** The top 10 most frequently used V-J gene combinations at the different time points for sequences determined by NGS. **e** Heavy chain CDR3 aa lengths at different time points

Peripheral blood mononuclear cells (PBMCs) and plasma samples were collected from individuals receiving an aerosolized Ad5-nCoV prime vaccination and an intramuscular Ad5-nCoV boost dose (Supplementary Fig. 2a and Supplementary Table 1). The aerosolized vaccine best follows the natural route of many infections.^18^ The IgG binding antibodies were robustly increased at 1-month post-prime vaccination (Supplementary Fig. 2b), and the 50% inhibitory concentration (IC_50_) were boosted by 5.8-, 4.9-, and 3.8-fold on average for the Wuhan-Hu-1, Beta, and Delta variants, respectively, from the prime dose to the second vaccine dose (Supplementary Fig. 2c). Blood samples from donor 3, with the highest neutralization titers of plasma IgG, were chosen for longitudinal analysis to monitor the induction and maintenance of antibody responses to vaccination against SARS-CoV-2 variants. The levels of the S protein-specific plasma IgG peaked at half a month after the boost dose and subsequently declined over a 6-month course (Fig. 1a). The S_ECD_-binding IgG concentration of the 50% of maximal effect (EC_50_) was increased by 3.2-fold for variants from 1-month post-prime vaccination to half a month post-boost, whereas the immunoglobulin A (IgA) EC_50_ value changed by only 1.1-fold during the same period (Supplementary Fig. 2d). Moreover, the neutralizing activities of plasma against the pseudotyped virus and authentic SARS-CoV-2 variants also peaked at half a month after the boost vaccination and declined over the next 6 months, but remained detectable at 6 months post-boost (Fig. 1b, c).

To characterize the genetic immune features after Ad5-nCoV vaccination, we performed next-generation sequencing (NGS) analysis of the overall B cell repertoires. Striking increases in IGHV2, IGHD6, and IGHJ5 were detected at 1 month after the aerosolized prime vaccination, whereas the variable gene family distribution after the intramuscular boost dose remained nearly unchanged (Supplementary Fig. 3a). A significant preference for the combination of IGHV2-5 and IGHJ4 in heavy chains was observed 1-month post-prime vaccination (Fig. 1d). The boost dose resulted in a minimum complementarity-determining region 3 (CDR3) length of an average of 16.8 amino acids (aa) at half a month post-boost (Fig. 1e). The extent of somatic hypermutation (SHM) increased after the second dose of vaccine (Supplementary Fig. 3d). The variable gene family distribution, the combination of IGLV and IGLJ, SHM, and the CDR3 length for light chains did not significantly change throughout the whole observation period (Supplementary Fig. 3b–e).

### Isolation of S protein-specific human mAbs

**Fig. 2:**
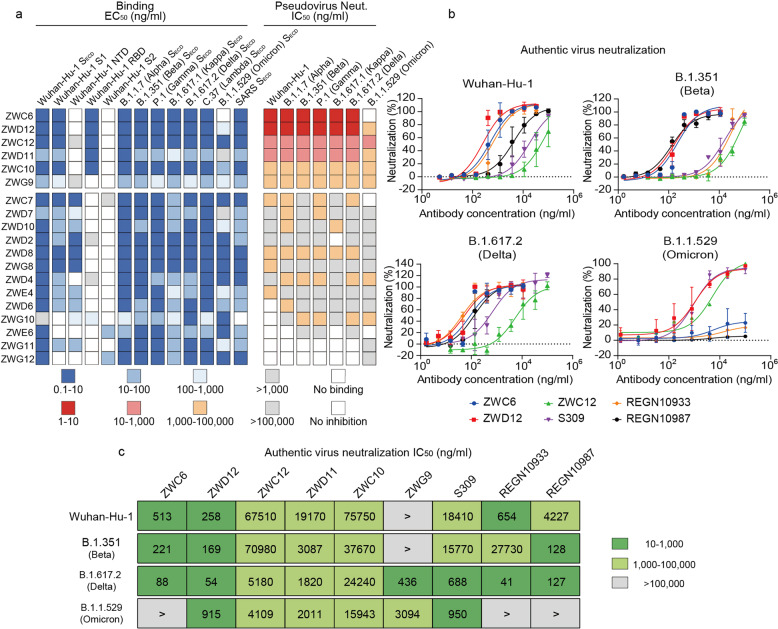
Neutralization of pseudotyped and authentic SARS-CoV-2 variants. **a** Heatmap showing the binding EC_50_ values for S fragments of SARS-CoV-2 variants and neutralization IC_50_ values of pseudotyped SARS-CoV-2 variants. **b** Neutralization curves of authentic SARS-CoV-2 variants by ZWC6, ZWD12, ZWC12, and FDA-approved mAbs. The data are representative of one of at least two independent experiments and are presented as the mean ± SD. **c** IC_50_ values of authentic virus-neutralizing mAbs. ND, not determined

To decipher Ad5-nCoV vaccine-induced antibody responses against SARS-CoV-2 variants at a molecular level, we performed single-B cell sorting from the PBMCs collected at half a month after the boost dose. The S_ECD_ protein of Wuhan-Hu-1 was used to probe the IgG^+^ and antigen-specific memory B cells at a frequency of 0.031%. We isolated 19 mAbs that could bind to Wuhan-Hu-1 S_ECD_, among which 78.9% (15/19) were reactive to S1 and 15.8% (3/19) targeted S2. Among the 15 anti-S1 mAbs, 80% (12/15) bound to the N-terminal domain (NTD), and 53.3% (8/15) interacted with the receptor-binding domain (RBD) (Fig. 2a). To determine the cross-binding abilities of the 19 mAbs, we assayed the binding EC_50_ values of these mAbs to the S_ECD_ of SARS-CoV-2 variants and SARS. We found that all 19 mAbs cross-reacted with the S_ECD_ of Alpha, Beta, Gamma, Kappa, Delta, and Lambda but not to that of Omicron, in which ZWC6 lost its ability to bind the Omicron S_ECD_ (Fig. 2a).

To measure the functional activities of the 19 binding mAbs (bAbs), we performd a neutralization assay on pseudotyped virus expressing the S protein of either the Wuhan-Hu-1, Alpha, Beta, Gamma, Kappa, Delta, or Omicron. We found that 52.6% (10/19), 63.2% (12/19), 47.4% (9/19), 57.9% (11/19), 42.1% (8/19), 52.6% (10/19), and 36.8% (7/19) of the mAbs neutralized the pseudoviruses of Wuhan-Hu-1, Alpha, Beta, Gamma, Kappa, Delta, and Omicron, respectively, with IC_50_ values below 100,000 ng/ml (Fig. 2a). For the neutralization assay against authentic virus, ZWD12 exhibited ultrapotent neutralizing abilities against Wuhan-Hu-1, Alpha, Beta, Gamma, Kappa, and Delta, whereas the control mAbs REGN10933 and REGN10987,^19^ which have been granted Emergency Use Authorization (EUA) by the Food and Drug Administration (FDA) were less able to neutralize Beta and Wuhan-Hu-1, respectively. Another approved antibody S309 (the parental form of sotrovimab, which is formerly known as VIR-7831) were also less able to neutralize Wuhan-Hu-1, Beta, and Delta compared with ZWD12. ZWD12 exhibited the highest neutralizing levels against authentic Omicron virus, with a slightly lower neutralizing IC_50_ compared with S309,^20^ whereas ZWC6, REGN10933, and REGN10987 failed to neutralize authentic Omicron variant (Fig. 2b, c).

### Characterization of nAbs

**Fig. 3:**
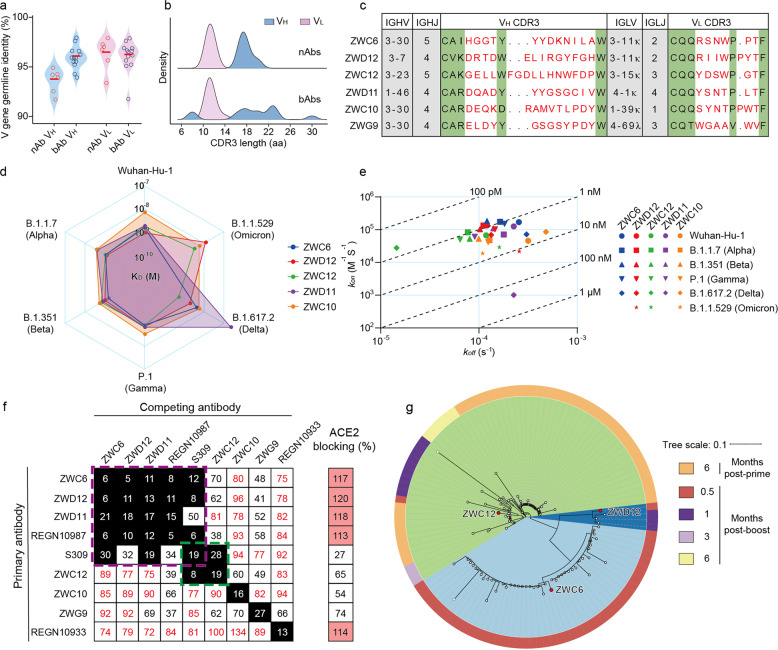
Binding properties of S protein-specific mAbs. **a** The V_H_ and V_L_ gene identities from the germlines of nAbs and bAbs. **b** CDR3 aa lengths of V_H_ and V_L_ of nAbs and bAbs. **c** The V_H_ and V_L_ gene usage, J gene usage, and CDR3 aa sequences of nAbs. **d** Binding kinetics (*K*_D_ values) of nAbs against the S proteins of SARS-CoV-2 variants as determined by SPR. **e** *K*_on_ and *K*_off_ of nAbs against the S proteins of SARS-CoV-2 variants as determined by SPR. **f** Heatmap showing the competing matrix of nAbs and the ability of mAbs to block the ACE2 and S-protein interaction determined by BLI. The numbers in the box indicate the percentage of binding of the competing mAb following the binding by primary antibody. The mAbs were considered competing if the inhibition percentage was <30% (black boxes with white numbers). The mAbs were deemed to be non-competing for the same site if the percentage was >70% (white boxes with red numbers). The white boxes with black numbers indicate an intermediate phenotype (30 to 70%). **g** The phylogenetic tree graph shows clones from the clonotype of ZWC6, ZWD12, and ZWC12 sequenced at different time points

NAbs demonstrated lower V_H_ germline identities (average of 93.6%) than bAbs (average of 95.9%), whereas V_L_ exhibited similar SHM levels, with germline identities of 96.3% and 96.1% for nAbs and bAbs, respectively (Fig. 3a). Moreover, the bAbs exhibited a broader V_H_ CDR3 length distribution than the nAbs (Fig. 3b). The combination of IGHV and IGHJ gene usage of the ZWC12, ZWC10, and ZWG9 antibodies dropped in the top 10 most frequently used combinations in the overall B cell repertoires (Figs. 3c and 1d). To characterize the interactions between the S_ECD_ of VOCs and nAbs, we determined the binding kinetics using surface plasmon resonance (SPR). ZWD12, ZWC12, and ZWC10 presented high binding affinities to the Wuhan-Hu-1, Alpha, Beta, Gamma, Delta, and Omicron S_ECD_, whereas ZWC6 and ZWD11 hardly bound to the Omicron S_ECD_ in SPR assay. The binding affinities of ZWG9 to Wuhan-Hu-1 and all 5 variants were very low (Fig. 3d, e and Supplementary Fig. 4). We assessed the competition between the nAbs and the other three control mAbs. ZWC6, ZWD12, ZWD11, REGN10987, and S309 fell into a group that competed with each other (Fig. 3f and Supplementary Fig. 5), and they all blocked S protein binding to ACE2 except S309. ZWC12 and S309 fell into the same group. To investigate the evolutionary trajectories of nAbs at different time points, we searched for the heavy-chain clonotypes with the identical V genes and similar (identical CDR3 amino acid length and five or less differences in absolute amino acid composition of CDR3s were considered as similar) CDRH3 in the B cell receptor (BCR) sequences at 7 time points. The ZWD12 clonotype was found only at 0.5 and 1-month post-boost, and the ZWC12 clonotype was found at 6 months post-prime and at 0.5, 1, 3, and 6 months after the boost dose; on the other hand, the clonotype of ZWG9, the nAb with the lowest binding affinities, was found at all 7 time points (Fig. 3g and Supplementary Fig. 6).

### ZWD12 and ZWC6 protect against challenge in the K18-hACE2 mouse model

**Fig. 4:**
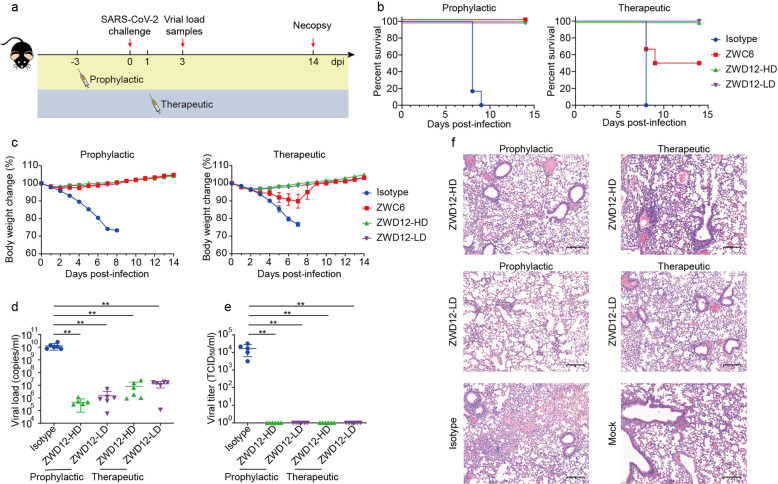
Prophylactic and therapeutic efficacy of ZWC6 and ZWD12 in the K18-hACE2 transgenic mouse model of SARS-CoV-2 infection. **a** Prophylactic and therapeutic study schema. **b** Survival rates of K18-hACE2 transgenic mice (*n* = 6) in the prophylactic and therapeutic groups. Isotype, a control antibody-targeted anthrax protective antigen. ZWD12-HD, treatment with ZWD12 at a high dose of 10 mg/kg. ZWD12-LD, treatment with ZWD12 at a low dose of 2 mg/kg. **c** Body weight changes of mice in the prophylactic and therapeutic groups. The mean ± SD are shown (*n* = 6). **d** Virus copy numbers in the lungs on day 3. **P < 0.01. **e** Viral titers in the lungs on day 3. **P < 0.01. **f** Histopathological changes in the lungs of SARS-CoV-2-infected mice collected at day 3. Scale bars, 200 μm

K18-hACE2 transgenic mice (6–8 weeks old) were infected with 2 × 10^3^ PFUs of the SARS-CoV-2 Delta variant (Fig. 4a). All mice in the control group died within 10 days post-challenge, accompanied by a dramatic weight loss. All mice in the prophylactic group that received 10 mg/kg ZWC6 three days in advance were able to survive lethal challenge without obvious weight loss, while those in the therapeutic group were partially protected (Fig. 4b, c). The mice in the prophylactic and therapeutic groups administered 10 mg/kg or 2 mg/kg of ZWD12 showed complete survivals against lethal virus challenge and lost less body weight (Fig. 4b, c). The animals in both the prophylactic group and the therapeutic group administered ZWD12 showed significant reductions in virus copy numbers (Fig. 4d) and viral titers (Fig. 4e) in their lung tissues and remarkable improvement of pulmonary pathological damage caused by virus infection (Fig. 4f). The inhibition of virus replication and alleviation of histopathological damage were better in the prophylactic group than in the therapeutic group.

### Cryo-EM structural analysis of the complexes of nAbs with the S protein

**Fig. 5:**
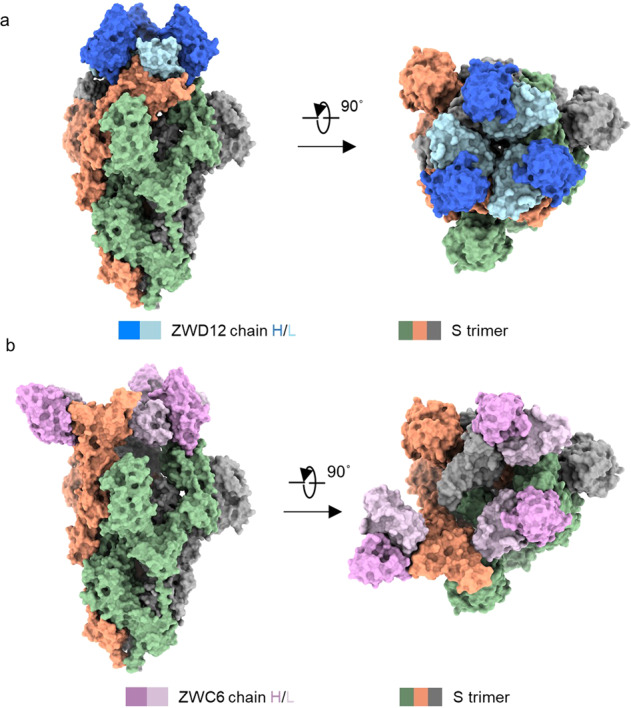
Cryo-EM complex structures of nAbs with the SARS-CoV-2 S protein. The domain-colored cryo-EM structures of the SARS-CoV-2 S protein in complex with ZWD12 (**a**) or ZWC6 (**b**) Fab, viewed along two perpendicular orientations. The heavy and light chains of ZWD12 and ZWC6 are colored blue, cyan, violet, and pink, respectively. The three protomers of the S protein are colored gray, orange, and green

To investigate the interactions of ZWC6 and ZWD12 with the S protein, we solved their cryo-EM structures in complex with S_ECD_ of the Delta variant at 2.8 Å and 3.0 Å resolution, respectively (Supplementary Figs. 7, 8 and Supplementary Table 2). Focused refinement was performed to improve the map quality at the binding interface between the RBD domain and ZWC6 or ZWD12, resulting in an improved local resolution of 3.5 Å and 3.3 Å, respectively, which allowed accurate model building for side chains of both nAbs and RBD. In the S_ECD_-ZWD12 complex, three Fabs molecules bound to the S protein in a closed conformation (Fig. 5a), whereas three ZWC6 Fab molecules bound to one “up” RBD and two “down” RBD in the S_ECD_-ZWC6 complex (Fig. 5b).

**Fig. 6:**
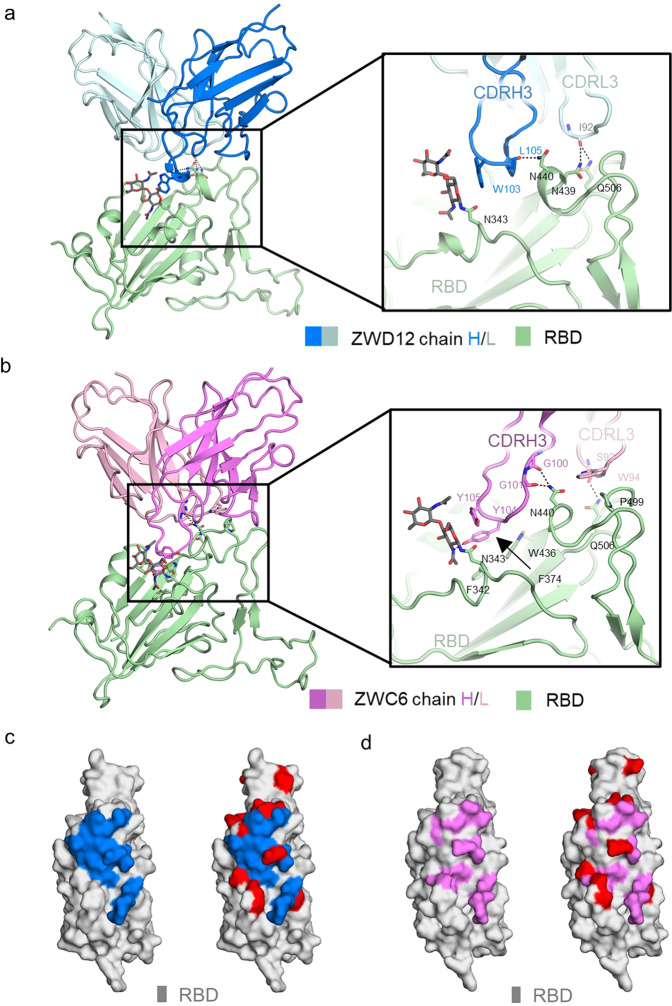
Interactions between nAbs and SARS-CoV-2 RBD. Binding interface between RBD and ZWD12 (**a**) or ZWC6 (**b**). The heavy and light chains of ZWD12 and ZWC6 are colored blue, cyan, violet, and pink, respectively. RBD is colored by green. The epitopes of ZWD12 (**c**) and ZWC6 (**d**) and the near Omicron variant mutations. ZWD12 and ZWC6 epitopes are colored blue and violet, respectively. Omicron variant mutations on RBD are shown in red

Detailed analysis shows that the interaction modes of ZWD12 and ZWC6 with the S protein are similar to REGN10987 and LY-CoV1404, which belong to the group D nAbs^21,22^ (Supplementary Fig. 9a, b). ZWD12 interacts with RBD mainly through its CDRH3 (residues 97 to 117) and CDRL3 (residues 90 to 104) loops in its heavy and light chains, respectively (Fig. 6a). The Trp103 residue of the heavy chain of ZWD12 approaches the glycosyl moiety on the N-glycosylation site of Asn343 of RBD. The main chain atoms of Leu105 in the heavy chain and the side chain of Ile92 in the light chain of ZWD12 also contribute to polar interactions with Asn440, Asn439, and Gln506 of RBD. Structural comparison shows that the ZWD12 and ZWC6 nAbs bound to RBD similarly (Fig. 6b and Supplementary Fig. 7a, c). The epitope of ZWD12 on the RBD contains Asn343, Trp436, Asn437, Ser438, Asn439, Asn440, Leu441, Val445, Pro499, Thr500, Asn501, Val503, Gln506, and Tyr508, and shows minimum overlap with the mutation sites of Omicron at only the N440K site, whereas ZWC6 epitope covers three Omicron mutation sites at S371L, S373P, and S375F (Fig. 6c, d), and the different sites between ZWD12 and ZWC6 may explain the broader neutralization ability of ZWD12 than ZWC6.

## Discussion

The Omicron variant has been rapidly spreading worldwide. Our results demonstrate that the plasma IgG and IgA induced by Ad5-nCoV vaccination against the Omicron S protein showed consistent levels of binding abilities to those of other SARS-CoV-2 variants. The neutralizing activity against Omicron was markedly improved after the first boost of the vaccine, despite the immune evasion of Omicron after the prime with the vaccine. A recent study reported that the efficacy of two-dose mRNA vaccine (BNT162b2) has reduced from over 90% against the original SARS-CoV-2 strain to ~40% against Omicron. Another study demonstrated that the plasma of subjects receiving 2 doses of BNT162b2 was 180-fold less potent against Omicron than Wuhan-Hu-1, and two-dose vaccinated individuals who received the third mRNA vaccine dose had increased in neutralizing activity against Omicron.^23^ Although the third immunization of BNT162b2 may not adequately protect against Omicron infection, these data combined with our data suggest the use of boosting as an optional further vaccination strategy to combat Omicron.

Omicron variant possesses extensive mutations in its S protein, raising concerns that the efficacy of current therapeutic antibodies might be compromised. Indeed, many mAbs clinically approved or in development, including LY-CoV016 (group A)/LY-CoV555 (group C),^24^ REGN10933 (group B)/REGN10987 (group D),^25^ AZD1061 (group D)/AZD8895 (group B),^26^ and BRII-196 (group A)^27^ showed substantially reduced neutralizing activity against the Omicron variant, while S309 (group E),^28^ DXP-604 (group A)^29^ and ADG-2^30^ were still functional with reduced and similar neutralizing capacities against Omicron.^22,31–33^ The group A-D nAbs target the S protein using overlapped epitopes with ACE2-binding motif, most of which lost or greatly reduced their neutralizing activity against Omicron. The group E-F nAbs, targeting more conserved epitopes outside the ACE2-binding motif, were less affected by Omicron.^11,34^ However, the mAb ZWD12 identified in this study belongs to the group D and exhibited ultrahigh neutralization activities against Alpha, Beta, Gamma, Kappa, and Delta variants; while it experienced a minor evasion of its neutralizing capacity by Omicron, its neutralizing efficacy was still comparable to S309, a member of group E, indicating that nAbs in group D can also be potential therapeutic antibodies against current VOCs and future emerging variants.

Besides, ZWD12 and S309 did not compete to the S protein binding, whereas REGN10987 and ZWC6 showed competing binding to the S protein with ZWD12. Interestingly, ZWD12 was able to overcome the Omicron’s antigenic shift, while ZWC6 and REGN10987 failed to neutralize Omicron. The different epitope sites on the S protein between ZWD12 with REGN10987 and ZWC6 may play critical roles in the immune evasion of Omicron variant. The structural characterization of ZWD12 and ZWC6 in our study could be used to the future rational design of vaccines broadly effective against SARS-CoV-2 variants.

Significantly lower mortality and less severe lung infection were recently reported in patients infected with the Omicron variant. The percentage of hospitalizations from the Omicron wave was also reported to be 41.3%, less than that seen in the Delta waves (about 68%).^35^ Lung infection and disease after Omicron infection was attenuated compared to Delta in animals.^36^ The Delta variant may still require continued therapeutic drug development, and ZWD12 still conferred the superior ability to provide neutralization against Delta. This work reports broad and potent human neutralizing mAbs and reveals their structural and functional basis for neutralization, providing a potent broadly protective mAb against highly transmissible SARS-CoV-2 VOCs and suggesting a promising approach to mitigate the risk of therapeutic resistance.

## Materials and methods

### Donor vaccination and blood sampling

Five healthy adults were vaccinated with the aerosolized adenovirus type-5 vector-based COVID-19 vaccine (Ad5-nCoV) and received boost immunization with Ad5-nCoV intramuscularly 3 months after the first vaccination. Blood samples, including plasma and PBMCs, were collected before both immunizations, at 1 or 6 months after the first immunization and at 0.5, 1, 3, and 6 months after the boost immunization. All donors provided written informed consent, and this study was approved by the Medical Ethics Committee, Academy of Military Medical Sciences (AMMS) with an approval number of AF/SC-08/02-147. PBMCs were separated from blood samples using Ficoll density gradient centrifugation (Tianjinhaoyang Biological Manufacture). Blood samples were slowly transferred above equal-volume lymphocyte separation medium. After centrifugation at 800 × *g* for 30 min, PBMCs were collected, washed twice with PBS, resuspended in cell freezing medium (90% FBS and 10% dimethyl sulfoxide (DMSO)), and stored at −80 °C until use.

### S protein expression and purification

For flowcytometry, ELISA, BLI, and SPR assays, genes encoding S_ECD_ (1–1208 a.a) of SARS-CoV-2 Wuhan-Hu-1 (GenBank ID: QHD43416.1), Alpha (GISAID ID: EPI_ISL_708969), Beta (GISAID ID: EPI_ISL_712081) and Gamma (GISAID ID: EPI_ISL_792680) variants as well as S_ECD_ (1–1190 a.a) of SARS-CoV (GenBank ID: YP_009825051.1) were codon optimized for human species and cloned into the pCAGGS vector. For SARS-CoV-2, “RRAR” at residues 682-685 was substituted with “GSAS” to abolish the cleavage site and two substitutions K986P and V987P were used to stabilize the prefusion post. A T4 fibritin trimerization motif followed by a polyhistidine tag, a twin-strep tag, and a FLAG tag were added at the C-terminus of the protein. The recombinant S_ECD_ protein was overexpressed using Expi293F mammalian cells and the ExpiFectamine™ 293 transfection kit (Gibco) at 37 °C under 5% CO_2_ in a Multitron-Pro shaker (Infors HT) according to the manufacturer’s protocol. The supernatant was collected at 4000 × *g* at 120 h post transfection and subjected to anti-FLAG affinity purification (Sigma Aldrich) according to the manufacturer’s protocol. Briefly, following the sample loading, the column was washed using the wash buffer (25 mM Tris, 150 mM NaCl, pH 8.0). The S protein was eluted using the elution buffer (25 mM Tris, 150 mM NaCl, 0.2 mg/mL FLAG peptide, pH 8.0). The buffer of the eluent was exchanged with 0.01 M phosphate-buffered saline (PBS, pH 7.2) and concentrated using a Centrifugal Filter Unit (Millipore). The final concentration of the protein was determined using NanoVue Plus (GE Healthcare) and the protein was then aliquoted and stored at −80 °C.

Recombinant S_ECD_ proteins of the SARS-CoV-2 Kappa (40589-V08B15), Delta (40589-V08B16), and Lambda (40589-V08B23) variants were purchased from Sino Biological. The recombinant S_ECD_ protein of the SARS-CoV-2 Omicron variant (SPN-C52Hz) was purchased from Acro Biosystems.

For cryo-EM sample preparation, we inserted the ECD (1–1208 a.a) of the spike protein of B.1.617.2 lineage Delta variant/2021 (GenBank ID: OK091006.1) into the pCAG vector (Invitrogen) with two proline substitutions at residues 986 and 987, a “GSAS” substitution at residues 682 to 685 and a C-terminal T4 fibritin trimerization motif followed by one Flag tag. Six proline substitutions that could stabilize the trimer spike were introduced at residues 817, 892, 899, 942, 986, and 987,^37^ and this construct was named S_ECD_-6p. The mutants were generated with a standard two-step PCR-based strategy.

The recombinant S_ECD_-6p protein was overexpressed in the Expi293F mammalian cells (Thermo Fisher Scientific) at 37 °C under 5% CO_2_ in a Multitron-Pro shaker (Infors, 130 rpm). When the cell density reached 2.0 × 10^6^ cells/mL, the S_ECD_-6p plasmid was transiently transfected into the cells. To transfect one liter of cell culture, ~1.5 mg of the plasmid was premixed with 3 mg of polyethylenimines (PEIs) (Polysciences) in 50 mL of fresh medium for 15 min before being added to the cell culture. Cells were removed by centrifugation at 4000 × *g* for 15 min after 60 h of transfection. The secreted S_ECD_-6P proteins were purified on an anti-FLAG M2 affinity resin (Sigma Aldrich). After loading two times, the anti-FLAG M2 resin was washed with wash buffer containing 25 mM Tris (pH 8.0) and 150 mM NaCl. The protein was eluted with wash buffer containing 0.2 mg/mL flag peptide. The eluent was then concentrated and subjected to size-exclusion chromatography (Superose 6 Increase 10/300 GL, GE Healthcare) in buffer containing 25 mM Tris (pH 8.0) and 150 mM NaCl. The peak fractions were collected and concentrated before incubation with a nAb. Purified S_ECD_-6P was mixed with the mAb at a molar ratio of approximately 1:5 for 1 h. Then, the mixture was subjected to size-exclusion chromatography (Superose 6 Increase 10/300 GL, GE Healthcare) in buffer containing 25 mM Tris (pH 8.0) and 150 mM NaCl. The peak fractions were collected for EM analysis.

### Antigen-specific B cell sorting

PBMCs were stained with anti-IgG-PE (BD), anti-CD19-Alexa Fluor700 (Beckman Coulter), anti-CD27-PE-Cy7 (BD) antibodies, and anti-CD3-PerCP (BD) as well as with the recombinant S_ECD_ of SARS-CoV-2 (Wuhan-Hu-1) at 4 °C for 1 h, followed by a two-step wash with PBS containing 2% FBS. The cells were then stained with anti-FLAG tag-FITC (Thermo Fisher Scientific) and anti-His tag-APC (Biolegend) at 4 °C for 1 h. After washing twice with 2% FBS-PBS, B cells were sorted using MoFloXDP cell sorter (Beckman Coulter). Target cells were defined as CD3^−^CD19^+^IgG^+^CD27^+^ S_ECD_^+^ and sorted as single cells into 96-well plates containing RNase-free water and RNase inhibitor (Promega). The plates containing cells were frozen using liquid nitrogen and then stored at −80 °C.

### Antibody preparation

Double-stranded cDNAs of the antibody V_H_ and V_L_ genes were produced in the sorted-cells plates using a One-Step reverse transcription PCR (RT-PCR) kit (Qiagen) and then amplified by nested PCR using TransStart Taq DNA polymerase (TransGen Biotech) as previously described.^38^ The PCR products were sequenced at Sangon Biotech, and the sequences were analyzed using IMGT/V-QUEST (http://www.imgt.org/IMGT_vquest). Linear expression cassettes were used to quickly express the antibodies. To produce heavy and light chain linear expression cassettes, the cytomegalovirus (CMV) promoter, Ig leader sequence, V_H_/V_L_ gene, Ig constant region (IgG1), and poly(A) sequence fragment were assembled using overlapping PCR as previously described.^39^ The full-length antibody sequences were cloned into pcDNA3.4. The plasmids of paired heavy and light chain genes were cotransfected into the Expi293F cells (Thermo Fisher Scientific) according to the manufacturer’s protocol. Antibodies were purified from cell culture supernatants using a HiTrap rProtein A column (Cytiva).

### Enzyme-linked immunosorbent assay (ELISA)

Antigen proteins (2 μg/mL) were added to ELISA microplates (Corning) and incubated overnight at 4 °C. After washing with PBS containing 0.2% Tween-20 (PBST), followed by blocking with 2% BSA in PBST at 37 °C for 1 h. Following washing with PBST, plasma samples or monoclonal antibodies were added to the plates and incubated at 37 °C for 1 h. Plasma samples were tested at a 1:40 starting dilution and at 7 additional threefold serial dilutions. Monoclonal antibodies were assayed at a 1 μg/mL starting concentration and at 7 additional fourfold serial dilutions. For plasma samples, plates were washed with PBST and then incubated at 37 °C for 1 h with an anti-human IgG, IgM, or IgA secondary antibody conjugated to horseradish peroxidase (HRP) (Abcam) in PBST at a dilution of 1:10,000. For monoclonal antibodies, plates were washed and then incubated with an HRP-conjugated anti-human IgG secondary antibody at a dilution of 1:10,000. After washing, the TMB substrate was added to the plates and incubated for 6 min at room temperature, and the reaction was stopped using 2 M H_2_SO_4_. The absorbance was read at 450 nm/630 nm using a microplate reader (Spectra Max 190, Molecular Devices).

### Pseudotyped virus-neutralizing assay

Genes encoding the full-length spike proteins of SARS-CoV-2 Wuhan-Hu-1 (QHD43416.1), Alpha (EPI_ISL_708969), Beta (EPI_ISL_712081), Gamma (EPI_ISL_792680), Kappa (EPI_ISL_1360306), Delta (EPI_ISL_2029113), and Omicron (EPI_ISL_6640917) were human codon optimized and inserted into the pCAGGS vector. HEK293T cells were inoculated into cell dishes and allowed to grow overnight at 37 °C and 5% CO_2_. The HIV-vectored pNL4-3.Luc.R^-^E^-^ and spike protein-expressing plasmids were cotransfected into HEK293T cells using Turbofect (Thermo Fisher Scientific). Supernatants containing pseudotyped virus particles were harvested at 48 h, 60 h, and 72 h post-transfection, filtered through a 0.45-μm filter, aliquoted and stored at −80 °C. To perform the neutralizing assay, 50 μL of pseudotyped virus was incubated with 50 μL of threefold serially diluted sera (starting dilution at 1:20) or a monoclonal antibody (starting concentration at 100 μg/mL) in 96-well cell culture plates at 37 °C for 1 h followed by the addition of 2.5 × 10^4^ ACE2-293T cells in 100 μL of DMEM supplemented with 10% FBS into each well. The plates were incubated at 37 °C and 5% CO_2_ for 48 h. After incubation, 100 μL of the medium was removed, and 100 μL of Britelite plus reagent (Perkin Elmer) was added to each well and incubated for 2 min. After incubation, each well was mixed 10 times by pipetting, and 150 μL of the mixture was transferred to a white plate to measure the luciferase activity using a microplate reader (TECAN Spark).

### Authentic virus-neutralizing assay

All processes in this study involving authentic SARS-CoV-2 were performed in a biosafety level 3 (BSL-3) facility. The neutralizing activity of plasma or antibodies against authentic SARS-CoV-2 wild-type (Wuhan-Hu-1), Beta, and Delta lineage were assessed using a microneutralization assay as previously described.^40^ Briefly, 3 × 10^4^ Vero-E6 cells in 100 μL DMEM medium with 10% FBS were inoculated into each well of 96-well cell culture plates and grown overnight at 37 °C and 5% CO_2_. Plasma (starting dilution at 1:4) or antibodies (initial concentration at 100 μg/mL) were serially diluted threefold and mixed with 100 TCID_50_ of SARS-CoV-2, followed by incubation at 37 °C for 1 h. After discarding the cell culture supernatants, 200 μL of the virus-antibody (or plasma) mixture was added to the Vero-E6 cells and incubated with 5% CO_2_ at 37 °C for 3 days. The crystal violet was used to stain the cells for 30 min at room temperature, and the absorbance at 570 nm/630 nm was then measured. Cells without viruses or antibodies were used as blank controls, and cells with viruses but without antibodies were used as virus controls.

SARS-CoV-2 Omicron virus was isolated from throat swab of a patient from Hong Kong by Institute of Laboratory Animal Sciences, Chinese Academy of Medical Sciences (CCPM-B-V-049-2112-18). The authentic SARS-CoV-2 Omicron neutralization assay was performed to evaluate the blockage of virus attachment by antibodies with a plaque assay. In brief, Vero cells were seeded at 1.5 × 10^5^ per well in 24-well culture plates overnight. The antibody samples were threefold serially diluted in DMEM with 2.5% FBS. An equal volume including 300 PFU/ml SARS-CoV-2 was added, and the antibody-virus mixture was incubated at 37 °C for 1 h. Then, half of the mixture was added to a 24-well culture plate containing Vero cells. The cells infected with 150 PFU/ml SARS-CoV-2 only and those without the virus were used as the positive and uninfected controls, respectively. After the sample plates were incubated at 37 °C for 1 h and the antibody-virus mixture was removed, the Vero cell surface was overlaid with 1 ml of DMEM with 2.5% FBS plus 0.9% carboxymethyl cellulose for further incubation at 37 °C with 5% CO_2_ for 3 days. Plaques were clearly observed after 0.5% crystal violet staining.

### Surface plasmon resonance (SPR) assay

Antibody-antigen binding kinetics were determined by SPR technology using a Biacore T200 instrument (GE Healthcare). The antibody was diluted in HBS-EP + buffer (GE Healthcare) to a concentration of 0.5 μg/mL and then subjected to a Protein A chip at a flow rate of 10 μL/min for 60 s. The S_ECD_ of SARS-CoV-2 (Wuhan-Hu-1) or its variants (Alpha, Beta, Gamma, Delta, Omicron) was tested using serially diluted concentrations (100, 50, 25, 12.5, 6.25, 3.125, 1.5625, and 0.78125 nM) at a flow rate of 30 μL/min. The flow durations were 120 s for the association stage and 900 s for dissociation.

### Biolayer interferometry (BLI) competition assay

The S_ECD_ of SARS-CoV-2 (Wuhan-Hu-1) was biotinylated using EZ-Link Sulfo-NHS-LC-LC- Biotin (Thermo Fisher Scientific). The biotinylated S_ECD_ was diluted in PBS containing 0.02% Tween 20 to a concentration of 200 nM and then immobilized onto streptavidin biosensors (Gator) for 50 s at 400 rpm. After washing with buffer for 60 s, the biosensors were immersed in wells containing the primary antibody (100 nM) diluted in the same buffer for 300 s at 1000 rpm, followed by incubation with the secondary antibody (100 nM) for 300 s.

### B cell repertoire preparation and next-generation sequencing

Total RNA was extracted from the PBMC sample using TRIzol (Invitrogen) according to the user manual. 5’ RACE was performed with a SMARTer RACE cDNA Amplification Kit (Clontech), and the total RNA input was 1 μg. For the IgG, the PCR mixture contained: 3 μL of cDNA and 20 pmol 5’ and 3’ primers (we used UPM primers from the RACE kit as the 5’ primers and IgG-specific primers as the 3’ primers (containing the sequence “ATGGGCCCTTGGTGG”)), 10 μL of 5 × pfu buffer, 4 μL of 2.5 mM dNTPs, 2.5 U pfu Taq, and water to a final volume of 50 μL. PCR conditions were as follows: 95 °C for 4 min followed by 25 cycles of 94 °C for 30 s, 58 °C for 30 s, and 72 °C for 10 s. The reactions were then held at 72 °C for 5 min and cooled to 4 °C. A similar approach was used for IgK and IgL amplification (3’ primers contained the sequences “ACAACAGAGGCAGTTCCAG” and “GTGTGGCCTTGTTGGCTT”, respectively). IgG/IgK/IgL NGS libraries were constructed using the NEBNext Ultra DNA Library Prep Kit for Illumina (NEB). After quality control analysis on a Bioanalyzer High Sensitivity DNA chip (Agilent), the libraries were sequenced on the Illumina platform. For data analysis, Trimmomatic software was adopted to finish QC for raw reads with default parameters. After QC, reads with adapters were discarded, and the bases of reads with a quality score lower than 20 were also removed. The clean reads were processed by MIXCR software to identify clones and corresponding CDR sequences. In this process, MIXCR software utilized V, D, and J gene reference sequences from B cells. Clone and CDR sequence information was used for further analysis.

### Next-generation sequencing data analysis

The V, D, and J gene segments and junctional bases of the antibody sequences were annotated with standalone IgBLAST^41^ 1.15.0 and a sequence database of germline gene segments from the international ImMunoGeneTics information system (IMGT).^42^ The standalone IgBLAST blast-style tabular output was parsed by the igblast subcommand of MakeDb.py in the Immcantation/Change-O toolkit^43^ to generate the standardized tab-delimited database file on which all subsequent Change-O modules operate. Germline reconstruction (CreateGermlines.py) was also performed using the Immcantation/Change-O toolkit on all heavy chain V sequences. Further analyses were implemented with R.^44^ The relative abundances of V(D)J alleles, genes or families within groups were determined with the function countGenes from the Alakazam^45^ version 1.0.2R package. Such data were further visualized in R using the function geom_bar from the ggplot2 v3.3.3 package. Multiple amino acid physicochemical properties were obtained with the function aminoAcidProperties from the Alakazam version 1.0.2R package. The amino acid sequence lengths of the CDR3 region in these tables were further visualized in R using the functions geom_bar and geom_smooth from the ggplot2 version 3.3.3 package. Mutation frequencies in V genes were then calculated using the calcObservedMutations function from the Immcantation/SHazaM v1.0.2R package. Violin graphics were obtained using the ggpubr version 0.4.0 packages. V gene family pairing information of antibodiy heavy chain and light chain was processed using R, and graphs were obtained using the pheatmap v1.0.12 package in R v3.6.3. A TOP10 Sankey plot was generated using the functions geom_flow and geom_stratum from the ggplot2 version 3.3.3 package.

### In vivo animal challenge experiment

The K18-hACE2 transgenic mice (GemPharmatech Co. Ltd., China), aged 6–8 weeks, were housed in a biosafety level-3 facility and given ad libitum access to standard pellet feed and water. All infectious experiments were performed in accordance with the standard operating procedures of the approved biosafety level-3 facility and were approved by the Animal Experiment Committee of Laboratory Animal Center, Institute of Microbiology and Epidemiology, AMMS (approval number: IACUC-IME-2021-017). Mice were intranasally inoculated with 2 × 10^3^ PFU of the SARS-CoV-2 B.1.617.2 (Delta) variant^40^ after intraperitoneal anesthetization with sodium pentobarbital. The mice in the prophylactic groups were treated with ZWD12 (2 mg/kg or 10 mg/kg) and ZWC6 (10 mg/kg) 3 days before infection. The mice in the therapeutic group received the same doses of ZWD12 and ZWC6 as those in the prophylactic group 1 day after virus challenge. The mice in the control group were administrated the isotype mAb 5E11.^46^ The mouse weights and survival were recorded daily for 14 days post-infection. In addition, mouse lung tissues were collected at 3 days post-infection for the assessment of virus replication and histopathological changes.

Viral titers in the lung homogenates of sacrificed mice were determined by the routine TCID_50_ method,^47^ whereas viral RNA in lung tissues was quantified by real-time RT-PCR. Briefly, total RNA in TRIzol (Ambion)-lysed lung tissues was extracted and reverse-transcribed to cDNA by using a reverse transcription kit (Takara). Subsequently, the viral copies targeting the E gene of SARS-CoV-2 were measured by real-time quantitative PCR using the following probe and primers:^48^ E_Sarbeco_F: ACAGGTACGTTAATAGTTAATAGCGT; E_Sarbeco_P1: FAM ACACTAGCCATCCTTACTGCGCTTCG-BBQ; E_Sarbeco_R: ATATTGCAGCAGTACGCACACA. The lungs of sacrificed mice were immediately fixed in 10% neutral buffered formalin, sectioned at a thickness of 4 μm thickness, and stained with hematoxylin and eosin (H&E) to examine histopathology.^49^

### Cryo-EM sample preparation and data collection

Aliquots (3.3 μL) of the concentrated samples (about 2.5 mg/mL) were applied to glow-discharged holey carbon grids (Quantifoil, Au R1.2/1.3), blotted for 2.5 s or 3.0 s and flash-frozen in liquid ethane cooled by liquid nitrogen with Vitrobot (Mark IV, Thermo Fisher Scientific). The prepared grids were transferred onto a Krios (Thermo Fisher Scientific) operating at 300 kV equipped with a Gatan K3 detector and a GIF Quantum energy filter. Movie stacks were automatically collected using AutoEMation,^50^ with a slit width of 20 eV on the energy filter and a defocus range of −1.2 µm to −2.2 µm in super-resolution mode at a nominal magnification of 81,000×. Each stack was exposed for 2.56 s with an exposure time of 0.08 s per frame, resulting in a total of 32 frames per stack. The total dose rate was ~50 e^−^/Å^2^ for each stack. The stacks were motion-corrected with MotionCor2^51^ and binned 2-fold, resulting in a pixel size of 1.087 Å/pixel. Moreover, dose weighting was performed. The defocus values were estimated with Gctf.^52^

### Data processing

The data processing was performed as previously described.^53^ Briefly, particles of S_ECD_-6P in complex with ZWD12 or ZWC6 were selected from micrographs using Relion 3.0.6. After 2D classification, good particles were selected and transferred to cryoSPARC. After two cycles of heterogeneous refinement, the good particles were selected and subjected to non-uniform refinement, local CTF refinement, and local refinement, obtaining the 3D reconstruction of the overall structure, which was then processed using Relion, including 3D auto-refinement and post-processing to get a better overall map. The interface between the spike protein and the antibody was subjected to focused refinement with a suited mask. The particles for the bnAbs-RBD sub-complexes were combined, re-extracted, and 3D classified with Relion. Then good particles were selected and subjected to 3D auto-refinement and post-processing, resulting in the 3D reconstruction of the bnAbs-RBD subcomplex with improved quality. For the dataset of S_ECD_-6P in complex with ZWC6, the processed data was further transferred from Relion to cryoSPARC. These particles were subjected to non-uniform refinement, local CTF refinement, and local refinement, finally resulting in overall and local maps of better quality.

The resolution was estimated using the gold-standard Fourier shell correlation (FSC) 0.143 criterion with high-resolution noise substitution. Refer to the Supplemental Information, Supplementary Figs. 7, 8 and Supplementary Table 2 for details regarding the data collection and processing.

### Model building and structure refinement

The model building and structure refinement were performed as previously described.^53^ Briefly, to build the models of the complex of S_ECD_-6P with ZWD12 or ZWC6, the atomic model of the SARS-CoV-2 spike protein in complex with 4A8 (PDB ID: 7C2L) was used as template, which were molecular dynamics flexible fitted (MDFF) into the corresponding cryo-EM maps. A Chainsaw model of ZWD12 or ZWC6 was first obtained using 4A8 as the template, which was further manually adjusted according to the local cryo-EM map focused on the bnAbs-RBD subcomplex in Coot. The residues were manually checked and adjusted. Some fragments were not modeled due to the poor quality. Real space refinement was accomplished using Phenix, with secondary structure and geometric restraints applied. To control the model overfitting, it was refined against one of the two independent gold-standard half maps. Then, the refined model was compared with the other map. The statistics of data collection, data processing, and model building are presented in Supplementary Table 2.

### Quantification and statistical analysis

For ELISA assay, the EC_50_ values for plasmas and monoclonal antibodies were determined using four-parameter nonlinear regression (GraphPad Prism v8.3). For pseudotyped virus neutralizing assay, the inhibition percent was calculated by comparing the relative luminescence units to the blank control (cells without pseudotyped virus or antibody) and virus control (cells with pseudotyped virus but without antibody). IC_50_ was calculated using a three-parameter nonlinear regression (GraphPad Prism v8.3). For authentic virus neutralizing assay, percent neutralization was calculated as (Sample signals − Virus control signals)/(Blank control signals − Virus control signals) × 100%. Data were fitted using a three-parameter nonlinear regression (GraphPad Prism v8.3). For SPR assay, affinity values, including association rates (*K*_on_), dissociation rates (*K*_off_), and affinity constants (*K*_D_), were calculated using Biacore T200 Evaluation Software with 1:1 binding model. For BLI competition assay, the competition value was defined as the percentage of the shift of tested group during the second antibody contact compared to the shift of control group, where buffer was used as the first antibody.

## Supplementary information

SUPPLEMENTAL MATERIAL

## Data Availability

Reagents generated in this study are available from the Lead Contact with a completed Materials Transfer Agreement. Atomic coordinates and cryo EM maps of the S protein of SARS-CoV-2 in complex with ZWD12 (PDB: 7WWL; whole map: EMD-32869, antibody-epitope interface-focused refined map: EMD-32870) or ZWC6 (PDB: 7WWM; whole map: EMD-32871, antibody-epitope interface-focused refined map: EMD-32872) have been deposited to the Protein Data Bank and the Electron Microscopy Data Bank, respectively. The codes and R packages are available at https://rdrr.io. Nucleotide sequences of all SARS-CoV-2 neutralizing antibodies were deposited at GenBank (accession numbers OM305108-OM305119. NGS data will be shared by the Lead Contact upon request.
