# Supplementary material for: Broadly neutralizing antibodies against Omicron-included SARS-CoV-2 variants induced by vaccination
Source: Signal Transduct Target Ther. 2022 Apr 27;7:139. doi: 10.1038/s41392-022-00987-z (PMC9044386; doi:10.1038/s41392-022-00987-z)
Supplement: Supplementary file 1 — SUPPLEMENTAL MATERIAL [file 41392_2022_987_MOESM1_ESM.docx]

Supplementary Materials for

Broadly neutralizing antibodies against Omicron-included SARS-CoV-2 variants induced by vaccination

Xiangyang Chi^1,5^, Yingying Guo^2,5^, Guanying Zhang^1,5^, Hancong Sun^1,5^, Jun Zhang^1,5^, Min Li^3,5^, Zhengshan Chen^1,5^, Jin Han^1^, Yuanyuan Zhang^2^, Xinghai Zhang^4^, Pengfei Fan^1^, Zhe Zhang^1^, Busen Wang^1^, Xiaodong Zai^1^, Xuelian Han^3^, Meng Hao^1^, Ting Fang^1^, Jinghan Xu^1^, Shipo Wu^1^, Yi Chen^1^, Yingying Fang^3^, Yunzhu Dong^1^, Bingjie Sun^1^, Jinlong Zhang^1^, Jianmin Li^1^, Guangyu Zhao^3^*, Changming Yu^1^*, Qiang Zhou^2^*, Wei Chen^1^*

Correspondence to: Wei Chen (cw0226@foxmail.com); Qiang Zhou (zhouqiang@westlake.edu.cn); Changming Yu (yuchangming@126.com); Guangyu Zhao (guangyu0525@outlook.com)

**This PDF file includes:**

Supplementary Figures S1 to S9

Supplementary Tables S1 to S2

­­


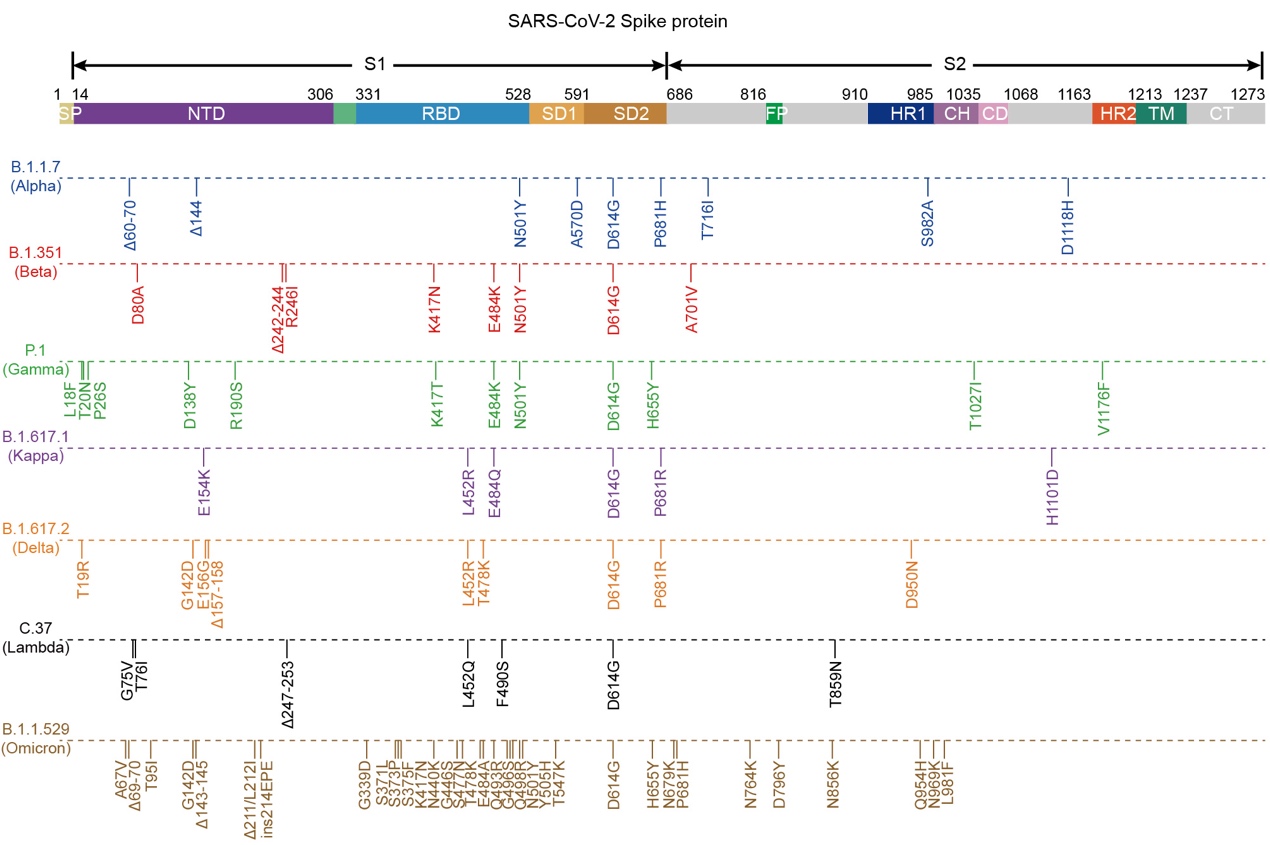


**Supplementary Figure S1. Schematic overview of SARS-CoV-2 variants.**

Schematic overview of the variants of concern B.1.1.7 (Alpha), B.1.351 (Beta), P.1 (Gamma), B.1.617.2 (Delta) and B.1.1.529 (Omicron) and the variants of interest B.1.617.1 (Kappa) and C.37 (Lambda). Amino acid modifications in comparison to the Wuhan-Hu-1 sequence are indicated.


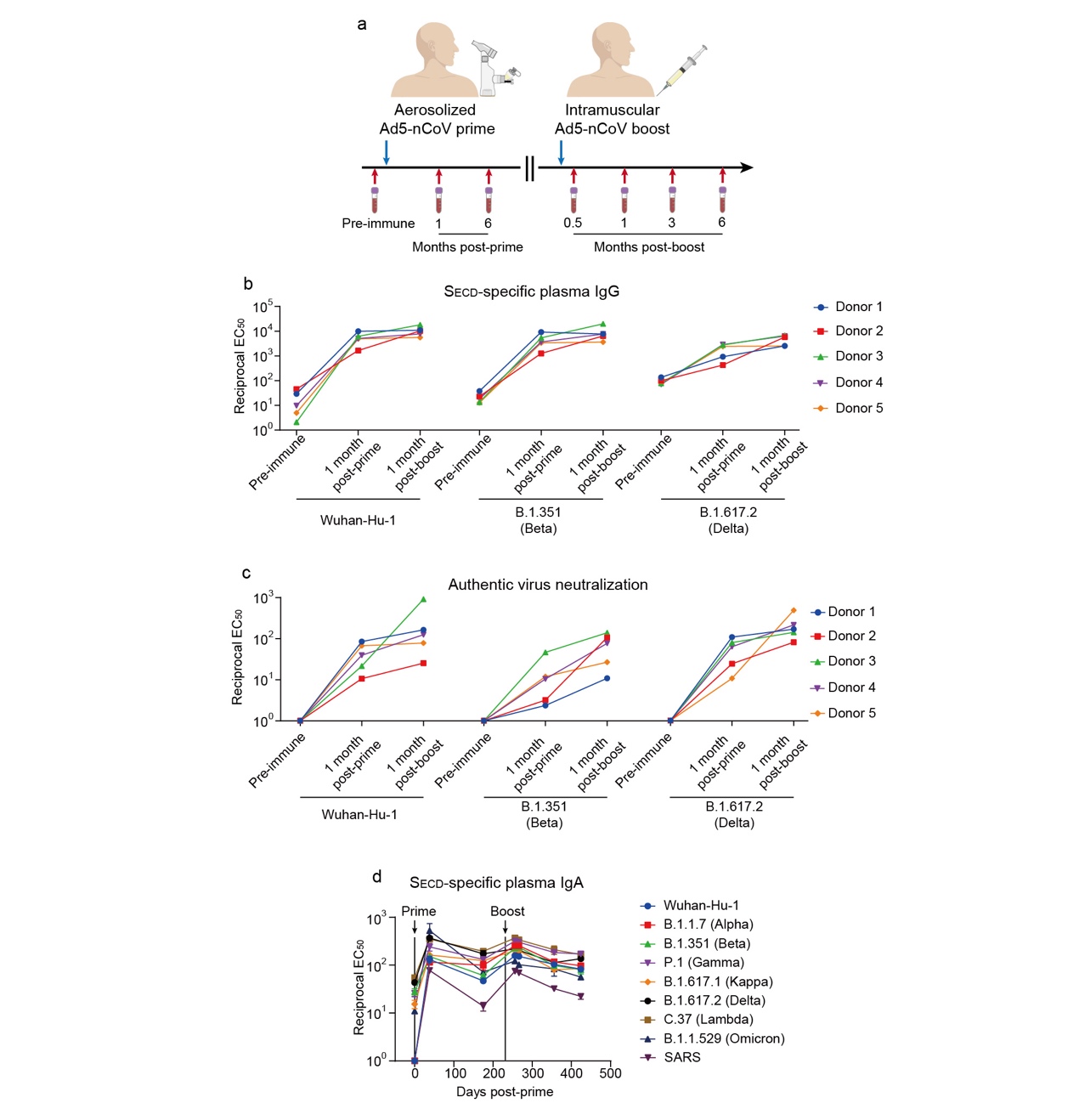


**Supplementary Figure S2. Polyclonal antibody responses to the Ad5-nCoV vaccine, related to Figure 1.**

**(a)** Study design. **(b)** EC_50_ titers of binding IgG antibodies against S proteins of different SARS-CoV-2 variants over time in plasma samples from five vaccinated individuals. **(c)** Authentic neutralization titers of SARS-CoV-2 variants in plasma samples from five vaccinated individuals. The data are represented as EC_50_ values. **(d)** EC_50_ titers of binding anti-S protein IgA antibodies over time in plasma samples from donor 3. The data are presented as the mean ± SD.


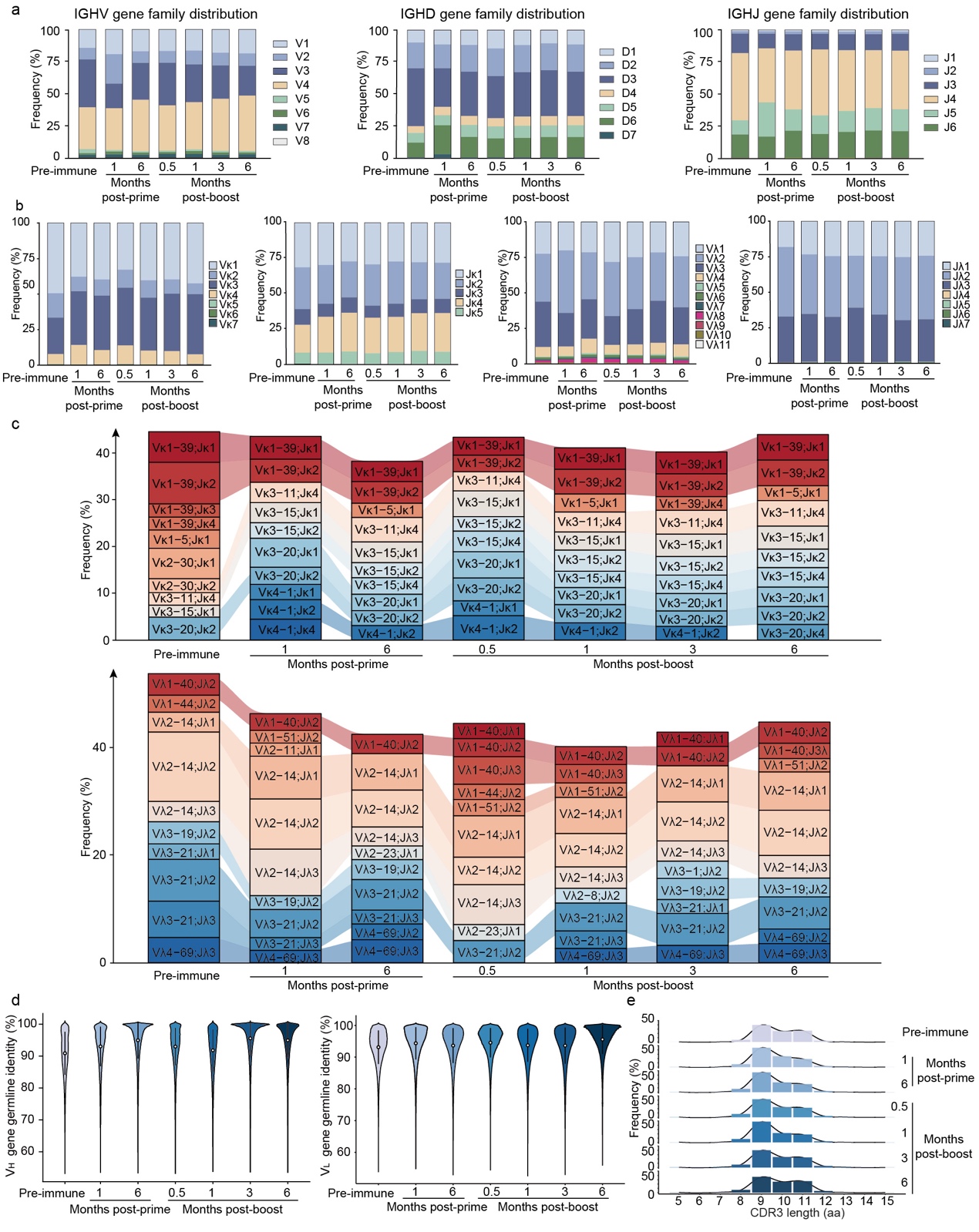


**Supplementary Figure S3. Gene analysis of B cell receptor as determined by next-generation sequencing, related to Figure 1.**

(**a**) V and J gene distributions for the heavy chain. Frequencies of V and J gene usage for the heavy chains. (**b**) V and J gene distributions for the light chain. Frequencies of V and J gene usage for the Kappa and Lambda light chains. (**c**) V_L_ gene and J_L_ gene combination. The top ten frequencies of V and J gene combinations for the Kappa chain and Lambda light chains. (**d**) Somatic hypermutation. Identity percentages of heavy chain and light chain variable genes to the germline. (**e**) Frequencies of the CDR3 amino acid lengths in the light chains.


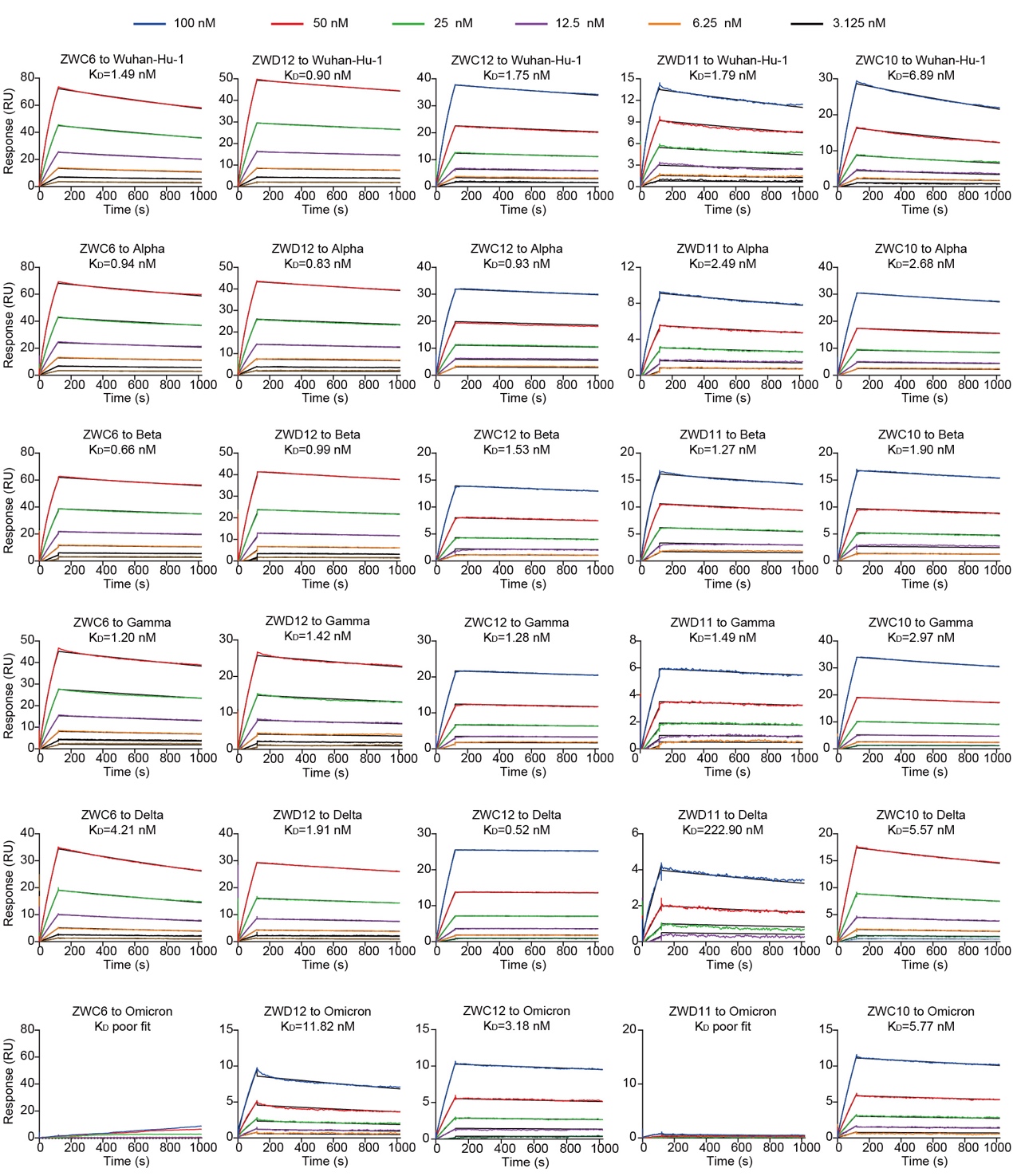


**Supplementary Figure S4. Surface plasmon resonance (SPR) affinity measurements, related to Figure 3.**

Binding kinetics curves of neutralizing antibodies to the S_ECD_ of different SARS-CoV-2 variants as determined using SPR.


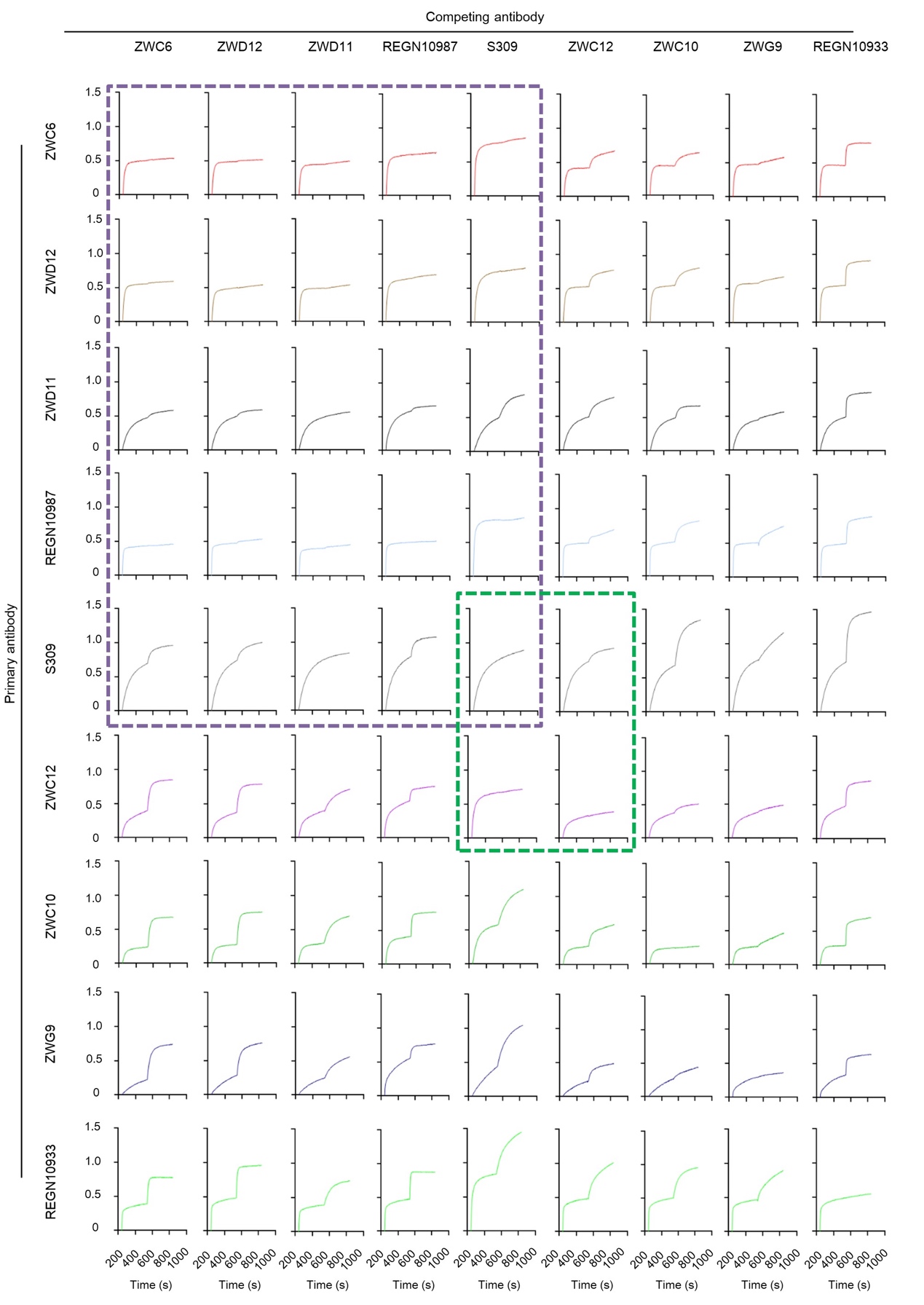


**Supplementary Figure S5. Biolayer interferometry competing experiment, related to Figure 3.**

Binding curves of the primary antibodies to the S protein followed by binding of the competing antibody.


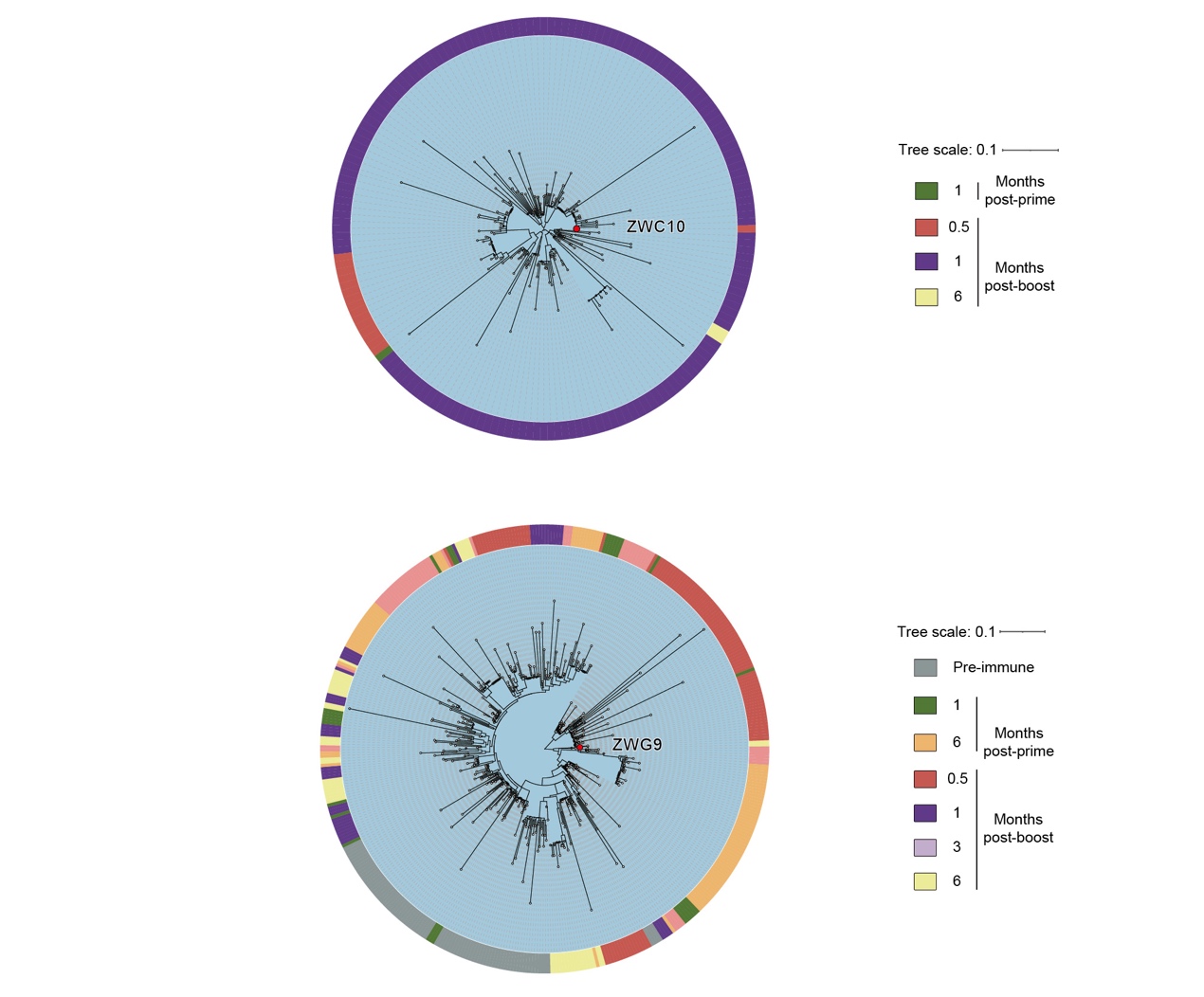


**Supplementary Figure S6. Clonal evolution of ZWC10 and ZWG8, related to Figure 3.**

The phylogenetic tree graph shows clones from the lineages of ZWC10 and ZWG9 sequenced at different time points.

**
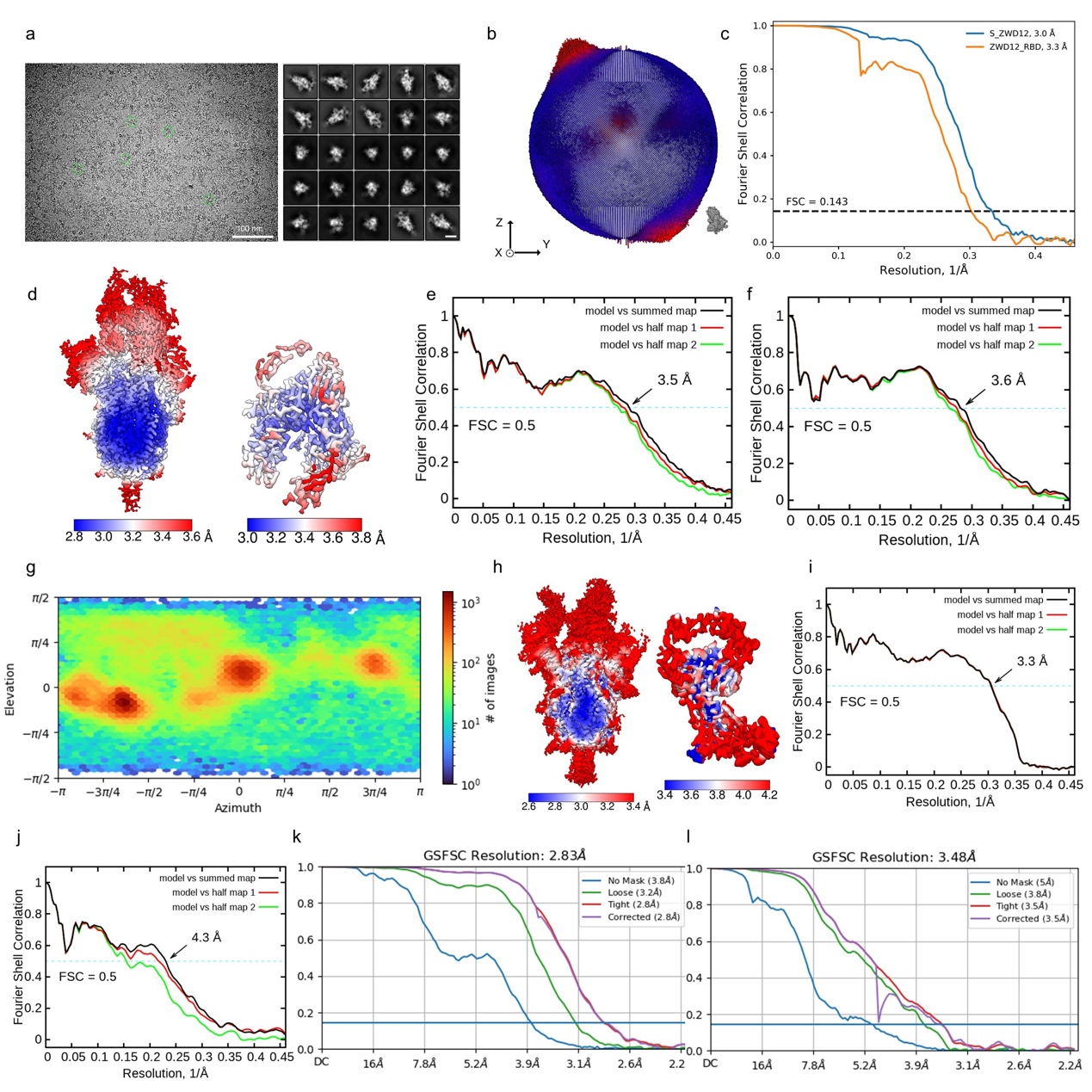
**

**Supplementary Figure S7. Cryo-EM analysis, related to Figure 5.**

**(a)** Representative cryo-EM micrograph and 2D class averages of cryo-EM particle images of S bound with ZWD12. The scale bar in 2D class averages is 10 nm. **(b)** Euler angle distribution in the final 3D reconstruction of S bound with ZWD12. **(c)** FSC curve of the overall structure (blue) and RBD-ZWD12 sub-complex (orange). **(d)** Local resolution map for the 3D reconstruction of overall structure and RBD-ZWD12 sub-complex, respectively. **(e)** FSC curve of the refined model of S bound with ZWD12 versus the overall structure that it is refined against (black); of the model refined against the first half map versus the same map (red); and of the model refined against the first half map versus the second half map (green). The small difference between the red and green curves indicates that the refinement of the atomic coordinates did not suffer from overfitting. **(f)** FSC curve of the refined model of RBD-ZWD12 sub-complex, which is same to the E. **(g)** Euler angle distribution in the final 3D reconstruction of S bound with ZWC6. **(h)** Local resolution map for the 3D reconstruction of overall structure and RBD-ZWC6 sub-complex, respectively. **(i)** FSC curve of the refined model of S bound with ZWC6 versus the overall structure that it is refined against (black); of the model refined against the first half map versus the same map (red); and of the model refined against the first half map versus the second half map (green). The small difference between the red and green curves indicates that the refinement of the atomic coordinates did not suffer from overfitting. **(j)** FSC curve of the refined model of RBD-ZWC6 sub-complex, which is same to the I. **(k-l)** FSC curve of the overall structure (blue) and RBD-ZWC6 sub-complex (orange).


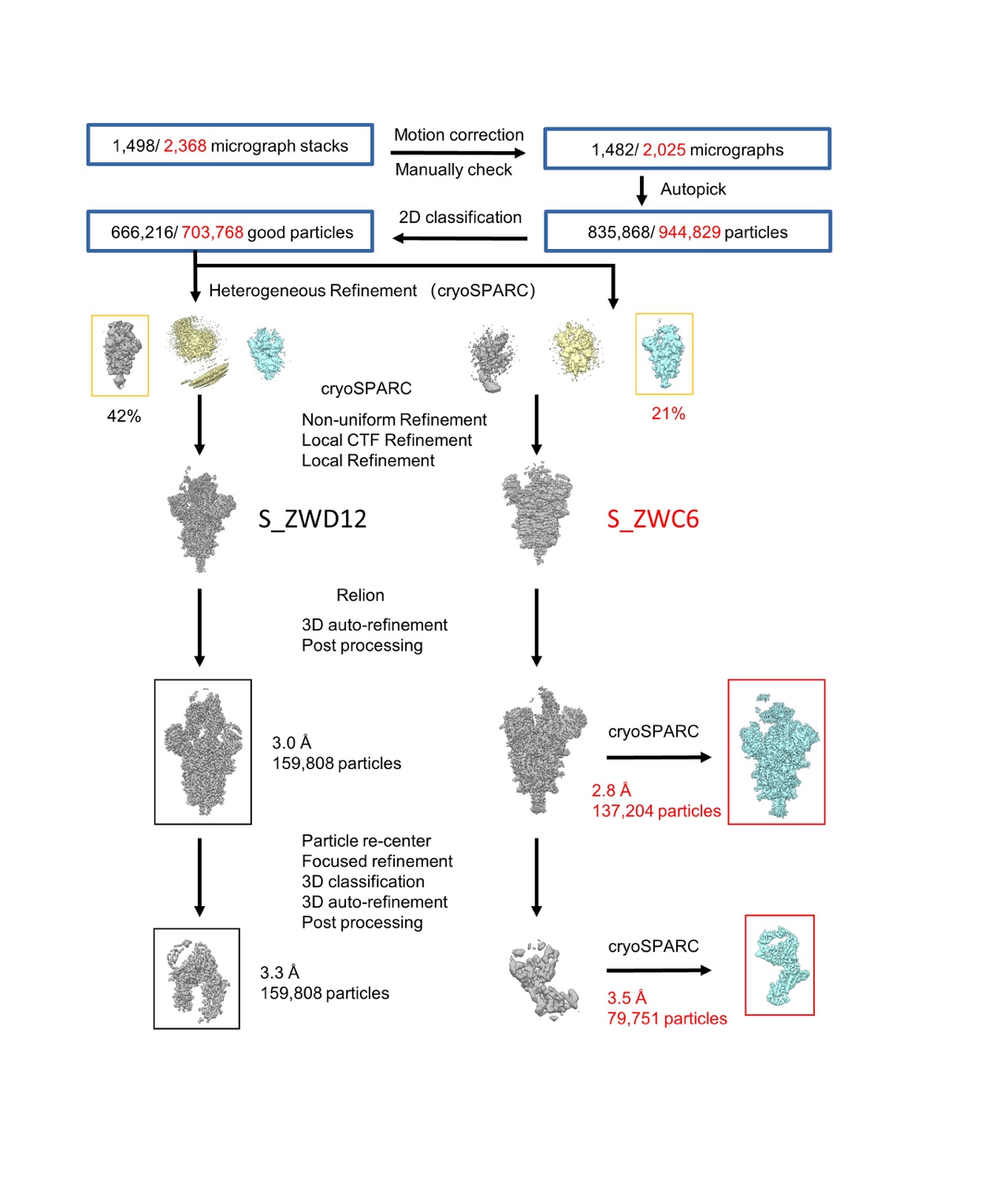


**Supplementary Figure S8. Flowchart for** **cryo-EM data processing and comparison of epitopes among multiple nAbs, related to Figure 5.**

Detailed cryo-EM data processing of S bound with ZWD12 and ZWC6.


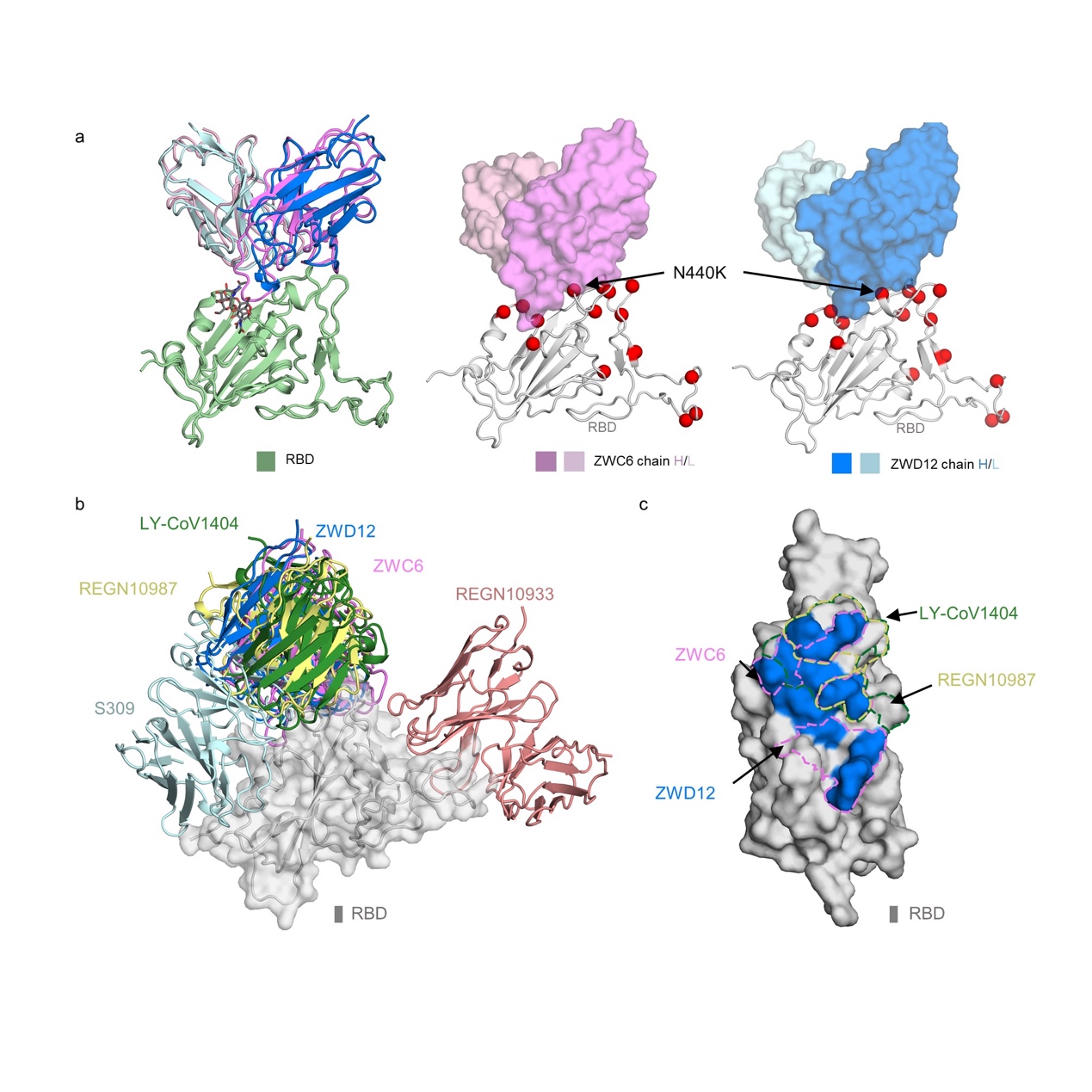


**Supplementary Figure S9. Structural comparison of ZWD12, ZWC6 and other bnAbs, related to Figure 6.**

**(a)** Comparison between ZWD12-RBD and ZWC6-RBD sub-complex. The heavy chain and light chain of ZWD12-RBD are colored blue and cyan, respectively. The heavy chain and light chain of ZWC6-RBD are colored violet and pink, respectively. The RBD is colored green. Omicron variant mutations of RBD domain are shown in red. **(b)** Comparison of binding modes of ZWD12, ZWC6, REGN10987, REGN10933, S309 and LY-CoV1404 which are colored by blue, violet, yellow, salmon, cyan and green, respectively. **(c)** Mapping of ZWD12, ZWC6, REGN10987 and LY-CoV1404 epitopes on RBD. The epitope surface of ZWD12 is blue. The borderlines of ZWD12, ZWC6, REGN10987 and LY-CoV1404 epitopes are shown in blue, pink, yellow and green, respectively.

**Supplemental Table 1 | Detailed information of the healthy donors**

| **Donor ID** | **Age (years)** | **Gender** | **Ethnicity** |
| --- | --- | --- | --- |
| 1 | 32 | Male | Chinese |
| 2 | 32 | Male | Chinese |
| 3 | 28 | Male | Chinese |
| 4 | 40 | Male | Chinese |
| 5 | 36 | Male | Chinese |

**Supplemental Table 2 | Data collection, 3D reconstruction and model statistic**

| **Data collection** |  |  |
| --- | --- | --- |
| EM equipment | Titan Krios (Thermo Fisher Scientific) | |
| Voltage (kV) | 300 | |
| Detector | Gatan K3 Summit | |
| Energy filter | Gatan GIF Quantum, 20 eV slit | |
| Pixel size (Å) | 1.087 | |
| Electron dose (e-/Å2) | 50 | |
| Defocus range (μm) | -1.2 ~ -2.2 | |
| Number of collected micrographs | 2,251 | |
| Number of selected micrographs | 1,982 | |
| Sample | S protein in complex with ZWD12 | |
| **3D Reconstruction** |  |  |
|  | Whole model | Interface between RBD and ZWD12 |
| Software | cryoSPARC/ Relion | Relion |
| Number of used particles | 349,830 | 316,510 |
| Resolution (Å) | 2.7 | 3.2 |
| Symmetry | C1 | |
| Map sharpening B factor (Å^2^) | -90 | |
| **Refinement** |  |  |
| Software | Phenix | |
| Cell dimensions (Å) | 313.056 | |
| Model composition |  |  |
| Protein residues | 4,572 | |
| Side chains assigned | 4,572 | |
| Sugar | 78 | |
| Linoleic acid | 3 | |
| R.m.s deviations |  |  |
| Bonds length (Å) | 0.007 | |
| Bonds Angle (˚) | 0.936 | |
| Ramachandran plot statistics (%) |  |  |
| Preferred | 93.61 | |
| Allowed | 6.16 | |
| Outlier | 0.23 | |
